# New genetic tools for central and peripheral vascular endothelia

**DOI:** 10.21203/rs.3.rs-7421061/v1

**Published:** 2025-08-27

**Authors:** Shangzhou Xia, Tenghuan Ge, Ruocen Song, Qinghai Liu, Xiao He, Yvonne Shen, Haowen Qiao, Yafei Qu, Jian-Fu Chen, Zhen Zhao, Xinying Guo

**Affiliations:** University of Southern California; University of Southern California; University of Southern California; University of Southern California; University of Southern California; University of Southern California; University of Southern California; University of Southern California; University of Southern California; University of Southern California; Guangzhou Women and Children’s Medical Center, Guangzhou Medical University

## Abstract

The divergence between the central and peripheral vascular system, particularly the emergence of the blood-brain barrier (BBB), is central to the brain’s homeostasis and functions. However, the molecular and genetic constituents that separating the BBB vascular cells from the rest remain elusive. Using single cell transcriptomics, we identified new cerebrovascular markers, e.g. zinc finger protein *Zic3* is explicitly found in adult brain endothelial cells and the *Atp13a5* ATPase is only expressed in brain pericytes. Using new genetic models, we further confirmed the specificity of *Zic3* in cerebrovasculature. Additionally, we developed a mouse model based on *Plvap*, and confirmed it is specific for endothelial cells of the peripheral tissue and circumventricular organs in brain. In-depth transcriptomics analysis between *Zic3*^*+*^ and *Plvap*^*+*^ endothelial cells revealed that genetic programs associated with lipid metabolism, transporter systems and tight junction signaling are critical drivers behind the separation of central and peripheral endothelia. These new murine genetic tools will further aid our understanding of vascular heterogeneity and BBB specialization.

The blood-brain barrier (BBB), blood-spinal cord barrier and blood-retinal barrier provide the physical barriers limiting the entrance of circulating pathogens and toxins, immune cells and body’s metabolic waste products into the central nervous system (CNS), and supplies critical energy metabolites such as glucose and lactate, essential amino acids, fatty acids, vitamins and growth hormones via selective transporter systems^1,2^. The BBB also helps clear brain’s own metabolic wastes including excess of neurotransmitters and proteinaceous molecules such as Alzheimer’s amyloid-β species, providing neurons with a tightly controlled microenvironment^3^. On the other hand, the vasculatures in the peripheral organs are more permeable, particularly in organs such as liver and kidney^4^. This functional heterogeneity also suggests that tissue microenvironment may provide important guidance cues for the specialization of their own vasculatures^5^. For example, Wnt signaling regulates both neural and BBB development in the vertebrate brain^6^, while hepatocyte growth factor (HGF) stimulates liver angiogenesis^7^.

Endothelial cells are the bedrocks of the vasculature. Anatomically, CNS endothelia are similar to the ones in the peripheral organs, connected by intercellular junctions and embedded in basement membrane. Yet, they are in close interactions with perivascular pericytes and astrocytic endfeet^8^, resulting in more tightly sealed tight junctions and minimal level of transcytosis^9^. At the molecular level, brain endothelial cells carry high levels of tight junctional proteins such as claudins and occludins^10^, and are enriched with special transporters such as glucose transporter 1 (GLUT1, encoded by *SLC2A1*)^10^, and major facilitator superfamily domain containing protein MFSD2A, a sodium-dependent lysophosphatidylcholine (LPC) transporter^11^. The peripheral endothelia on the other hand are often fenestrated^12^, providing non-selective transcellular transport through pinocytosis. The plasmalemma vesicle-associated protein (PLVAP), functioning as the diaphragm of these fenestrae, is in general absent in the adult brain^13^. However, the intrinsic programs underlying the central and peripheral divergence and the heterogeneity of vascular system remain underexplored. In addition, existing genetic tools (e.g., reporters and Cre drivers) are inadequate to differentiate or specifically manipulate central or peripheral vasculature endothelia, posing a significant research barrier.

To address these gaps, we analyzed single-cell transcriptomic datasets from 11 mouse tissues to identify endothelial markers with organ-specific expression patterns and revealed that Zic Family Member 3 (*Zic3*) is explicitly expressed in brain endothelial cells. We generated a *Zic3-T2A-tdTomato-IRES-CreERT2* knock-in model, with both fluorescent tdTomato reporter and CreERT2 recombinase expressed from the endogenous alleles, and confirmed both *tdTomato* reporter expression profiles and CreERT2 recombinase activity are specific to the endothelial cells of the central nervous system, including brain, spinal cord and retina. Furthermore, we generated a *Plvap-T2A-EGFP-IRES-CreERT2* knock-in model, and found that it reliably targets the peripheral endothelial cells in heart, lung, liver and kidney, as well as the endothelial cells in the choroid plexus and meningeal vessels in the brain. Based on these two markers, we further analyzed the endothelial transcriptomics at single-cell level, which revealed that transporter system and tight junction signaling are the major divergent points between the central and peripheral vasculatures. In sum, these new *Zic3* and *Plvap* genetic models represent unique tools towards a better understanding of the molecular and genetic underpinning of the complexity of our vascular system in development, aging and diseases.

## Results

### Differential vascular permeabilities between CNS and peripheral organs

**Figure 1:**
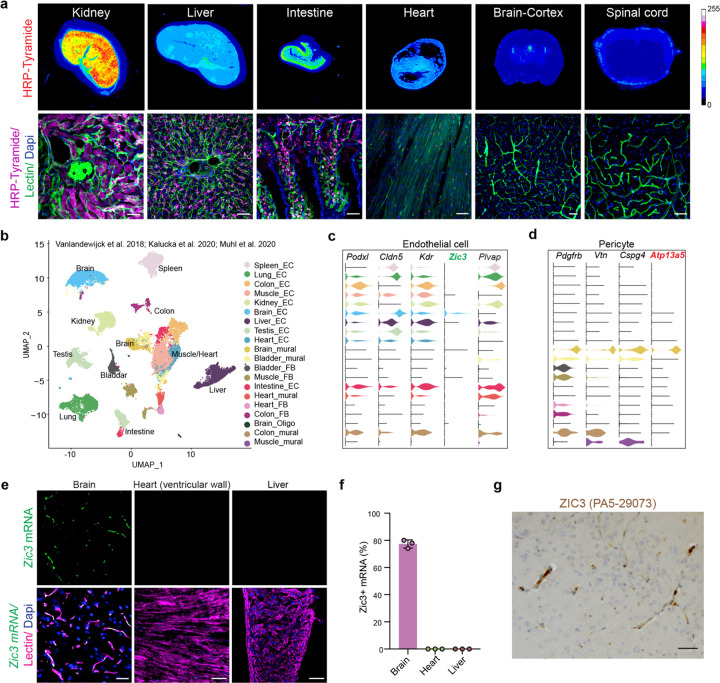
New cerebrovascular marker based on single cell transcriptomics. **(a)** Representative images of mouse kidney, liver, intestine, heart, brain, spinal cord following the intravenous injection of HRP. Scale bar: 50 μm. **(b)** UMAP of vascular cells transcriptomics from 11 tissues transcriptomes. Each dot was color-coded and annotated by both organs and cell types. EC: endothelial cell; mural: mural cell; FB: fibroblast; Oligo: oligodendrocytes. **(c)** Violin plots showing the distribution of expression level of the endothelial markers across 20 cell types. **(d)** Violin plots showing the distribution of expression level of the pericyte markers across 20 cell types. **(e)** Representative images showing RNAscope FISH for *Zic3* transcripts (Green), and immunostaining for Lectin^+^ endothelial cells (Magenta) and Dapi for nuclei (Blue) in the cortex of brain, the ventricular wall of heart and liver. Scale bar: 50 μm. **(f)** Percentage of *Zic3* mRNA-expressing cells in different mouse tissues. n = 3 mice. Data are presented as mean ± SEM. n = 3 mice. Data are presented in mean ± SEM. **(g)** Immunohistochemistry staining of paraffin-embedded mouse cortical sections using rabbit polyclonal anti-ZIC3 antibody (PA5–29073).

The divergence between the central and peripheral vascularsystems in terms of permeabilities has been known for more than a century^14^, since the early observations based on dye injections in embryos^15^. Using HRP as a traditional tracer administered in the circulation^16^, we compared the vascular permeabilities between the central and peripheral organs (**Extended Data Fig. 1a**). Briefly, mice received a single dose of 0.5 mg/g of body weight of HRP solution in saline, and tissues were harvested 2 h later after perfusion. With fluorescent Tyramide signal amplification (TSA)^17^, we can amplify the HRP tracer and compare the vascular permeabilities between different organs (Fig. 1a and **Extended Data Fig. 1b**). Among peripheral organs, kidney exhibited the highest vascular permeability, followed by liver, intestine and lung, while heart and spleen showed relatively low levels of HPR molecule accumulation. On the other hand, the brain and spinal cord had very minimal HRP signals, particularly in the cortex, or the grey matter of spinal cord (Fig. 1a).

### *Zic3* is a new marker for brain endothelial cells

To explore the molecular basis of vascular heterogeneity, we integrated and performed secondary analysis on three single cell RNA sequencing (scRNA-seq) datasets covering both mouse brain and peripheral vasculatures^18–20^. 48,526 single-cell transcriptomes were collected for the secondary analysis, as we recently reported^21^. In Uniform Manifold Approximation and Projection (UMAP), four major vascular cell types were separated into 20 clusters across different tissues including endothelial cells, mural cells, Oligodendrocytes, and fibroblasts (Fig. 1b), based on their specific genetic markers. These include but are not limited to *Cldn5*, *Podxl* and *Kdr* for endothelial cells, *Pdgfrb*, *Vtn* and *Cspg4* for mural cells. Interestingly, we found that *Zic3* is unique to the brain endothelium (Fig. 1c), and confirmed that *Atp13a5* is a brain mural cell marker^21^ (Fig. 1d).

*Zic3* encodes a zinc finger protein and transcription factor, essential for mammalian embryonic cardiovascular and neural development^22^. It is highly specific to the brain based on the Tabula Muris dataset^23^ (**Extended Data Fig. 1c-d**). To further confirm its specificity, we analyzed the brain vascular scRNA-seq dataset^18^ and found that *Zic3* expression is exceedingly precise to brain endothelial cells of capillaries, arterioles and veins, but not expressed in any other vasculature cells including VSMCs, oligodendrocytes, fibroblasts, microglia, or astrocytes (**Extended Data Fig. 1e**). In addition, analysis of a large dataset of 1,093,785 cells combining adult mouse cortical and hippocampal areas^24^ confirmed that *Zic3* expression is exclusively in endothelial cells, but not in any neuronal and glial cells (**Extended Data Fig. 1f**). Besides *Zic3*, we examined transporters that are currently known to be enriched in brain endothelial cells. For example, lysolipid transporter *Mfsd2a* and glucose transporter *Slc2a1* are well-known for their roles in cerebrovasculature^25,26^, but their expressions are evident in testicular endothelial cells based on the integrated transcriptomics (**Extended Data Fig. 1g**). In addition, although organic anion transporter *Slco1c1* is specific to brain endothelial cells in the integration dataset, it is highly expressed in astrocytes as well, based on datasets including the Tabula Muris^23^ (**Extended Data Fig. 1h**).

Fluorescent *in situ* hybridization (FISH) with RNAscope probes validated that *Zic3* transcripts are expressed throughout the brain regions including cortex, but not observed in peripheral organs such as liver or heart (Fig. 1e–f), and *Zic3* mRNA transcripts were colocalized exclusively within the lectin-positive brain endothelium (**Extended Data Fig. 2a**). In addition, immunohistochemistry analysis with antibodies that react with murine ZIC3 protein confirmed its presence in vasculature across brain regions (Fig. 1g), including cortex, hippocampus, cerebellum, hypothalamus, striatum, medulla, midbrain, thalamus and pons (**Extended Data Fig. 2b**). This indicates that *Zic3* is potentially a new specific marker for CNS endothelial cells.

### *Zic3-T2A-tdTomato-IRES-CreERT2* knock-in model for CNS endothelial cells

**Figure 2:**
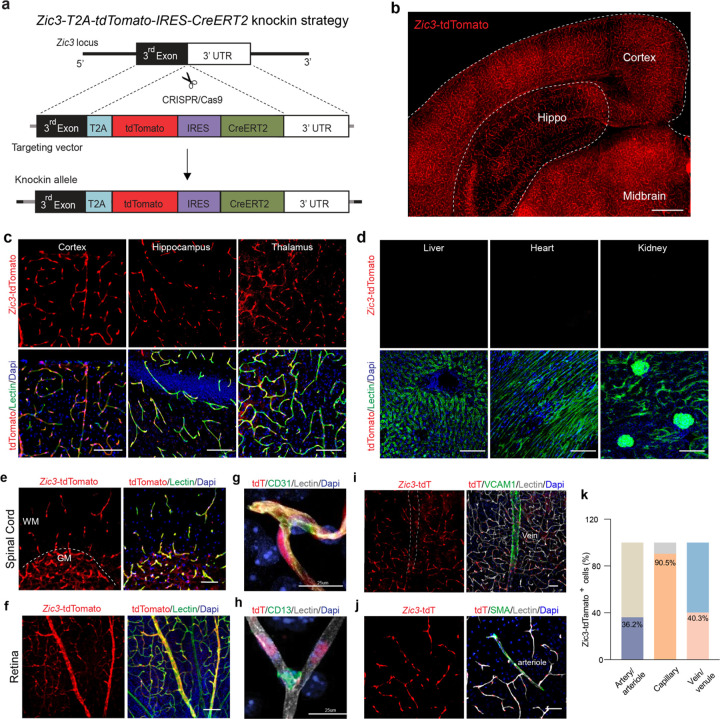
*Zic3* model for blood-brain barrier specific endothelial cells. **(a)** Schematic diagram showing the strategy for generating the *Zic3-T2A-tdTomato-IRES-CreERT2* knock-in mice. See Methods for more details. **(b)** A representative tiled image of brain section from a homozygous *Zic3-T2A-tdTomato-IRES-CreERT2* knock-in mouse. Scale bar: 500 μm. **(c)** Representative confocal images of *Zic3*-tdTomato (red), endothelial lectin (green), and DAPI (blue) in multiple brain regions from a homozygous *Zic3-T2A-tdTomato-IRES-CreERT2* mouse. Scale bar, 100 μm. **(d)** Representative confocal images of *Zic3*-tdTomato, endothelial marker Lectin (green) and Dapi (blue) in different tissues from a homozygous *Zic3-T2A-tdTomato-IRES-CreERT2* mouse, including liver, heart (ventricular wall) and kidney. Scale bar: 100 μm. **(e-f)** Representative confocal images showing *Zic3*-tdTomato expression, lectin (green) and Dapi (blue) in spinal cord and retina from homozygous *Zic3-T2A-tdTomato-IRES-CreERT2* mouse. Scale bar: 100 μm. **(g-h)** High magnification confocal images showing *Zic3*-tdTomato expression colocalized with CD31+ endothelial cell, but not CD13+ pericytes. Scale bar: 25 μm. **(i)** *Zic3*-tdTomato expression on brain capillary, and VCAM1^+^ vein/venules. Scale bar: 50 μm. **(j)** *Zic3*-tdTomato expression on brain SMA^+^ artery/arterioles. Scale bar: 50 μm. **(k)** The percentage of Zic3-tdTomato^+^ cells distributed among artery/arterioles, capillaries and vein/venules in the cortex. Arteries and arterioles are identified by vessel diameter in combination with the presence of SMA. Veins and venules are identified by vessel diameter in combination with the presence of VCAM1 and the absence of SMA. Lectin^+^ vessels with diameters smaller than 6 μm are considered as capillaries. n = 3 mice.

Next, we generated a new transgenic model targeting the *Zic3* locus. This *Zic3-T2A-tdTomato-IRES-CreERT2* knock-in model carries expressions of both tdTomato reporter and CreERT2 recombinase through the endogenous *Zic3* locus (Fig. 2a, also see Methods). One F0 founder was selected based on germline transmission and genome sequencing, and the F1 generation was further tested with southern blot analysis and validated by PCR (**Extended Data Fig. 3a-c**). As *Zic3* is located on X-chromosome, the model is maintained as hemizygous (*Zic3*^*tdT/Y*^ for male, and *Zic3*^*tdT/+*^ for female). Homozygous female *Zic3*^*tdT/tdT*^ mice can be obtained from breeding of hemizygous, and appear normal. Importantly, tdTomato is reliably expressed in adult hemizygotes and homozygotes (Fig. 2b).

The *Zic3-*tdTomato profiles overlay well with Lectin-labeled endothelium throughout the brain, including cortex, hippocampus and thalamus (Fig. 2c), but not in peripheral tissues such as liver, heart or kidney (Fig. 2d). To verify the expression of *Zic3* marker in the CNS, we also examined the spinal cord and retina in our transgenic model. We found robust *Zic3-*tdTomato signals in both white and gray matter of the spinal cord (Fig. 2e), with a little higher coverage in gray matter. In the retina, *Zic3-*tdTomato expressing cells were found in most of the vessels (Fig. 2f and **Extended Data Fig. 3d**).

Additional immunostainings further confirmed that *Zic3*-tdTomato expressing cells are indeed brain endothelial cells, as they are CD31-positive (Fig. 2g and **Extended Data Fig. 3e**), but not other cell types in the brain such as CD13-positive pericytes (Fig. 2h and **Extended Data Fig. 3f-g**), NeuN-positive neurons, GFAP-positive astrocytes, or IBA1-positive microglia (**Extended Data Fig. 3h-j**). *Zic3*-tdTomato reporter is found in over 90% of capillary endothelial cells, as well as in ~40% of endothelial cells on VCAM1-positive veins and venules or SMA-positive arteries and arterioles (Fig. 2i–k). It is estimated to cover at least 80% of the cerebrovasculature (**Extended Data Fig. 3k**).

### Characterization of the *Zic3*-CreER recombinase activity

**Figure 3:**
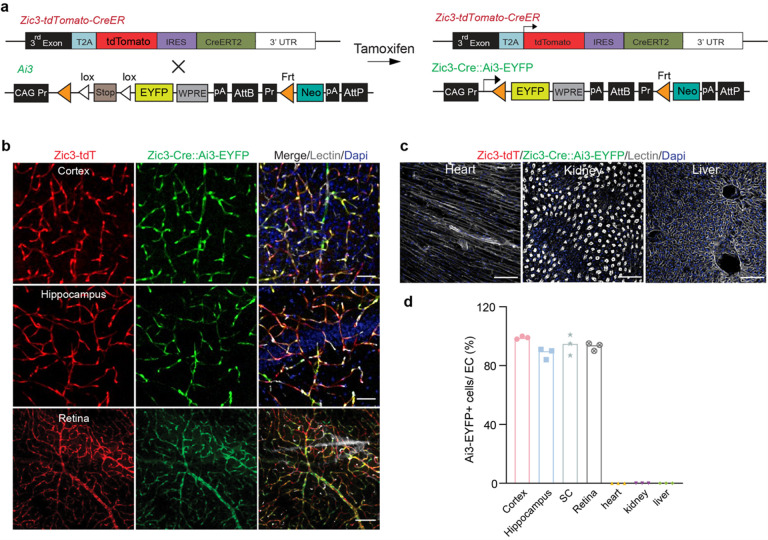
Additional characterization of Zic3-T2A-tdTomato-IRES-CreERT2 knock-in model. **(a)** Schematic diagram showing the breeding strategy for generating *Zic3-T2A-tdTomato-IRES-CreERT2; Ai3* mice. **(b)** Representative confocal images of *Zic3*-driven tdTomato (red), tamoxifen-induced EYFP (green), Dapi (blue) and lectin-labelled endothelial profiles (gray) in different tissues from a *Zic3-T2A-tdTomato-IRES-CreERT2; Ai3* mice, including cortex, hippocampus and retina. Scale bar: 100 μm. **(c)** Representative confocal images of heart, kidney and liver sections from *Zic3-T2A-tdTomato-IRES-CreERT2; Ai3* mice, showing no tdTomato or EYFP expression. Lectin (gray): endothelial profiles. Scale bar: 100 μm. **(d)** Quantification of the percentages of tdTomato^+^ and EYFP^+^ double positive cells in endothelia cells in different tissues as indicated. SC: spinal cord. n = 3 mice. Data are presented in mean ± SEM.

To test the CreER activity in our *Zic3-T2A-tdTomato-IRES-CreERT2* model, we crossed it with the Ai3-EYFP floxed reporter line^27^, and induced the CreER activity with tamoxifen administration (Fig. 3a, also see Methods). With 7 injections of Tamoxifen, more than 90% of *Zic3*-tdTomato^+^ endothelial cells in the brain, retina and spinal cord expressed robust EYFP signals (Fig. 3b–d). Yet, no recombination or EYFP reporter signal was observed in peripheral tissues such as heart, kidney or liver (Fig. 3c–d). Hence, our data demonstrated that *Zic3* marker is unique for the CNS endothelium, and *Zic3-T2A-tdTomato-IRES-CreERT2* model may help us decode the molecular and genetic underpinning of its specification.

### *Plvap-T2A-EGFP-IRES-CreERT2* model for peripheral and non-BBB endothelial cells

**Figure 4:**
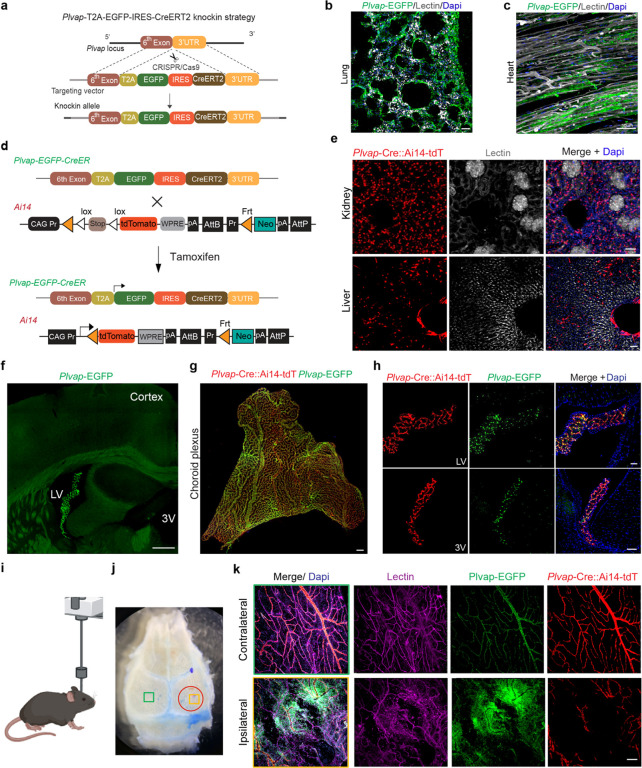
*Plvap* model for peripheral and non-BBB endothelial cells. **(a)** Schematic diagram showing the strategy for generating the *Plvap-T2A-EGFP-IRES-CreERT2* knock-in mice. See Methods for more details. **(b)** Representative image of fixed frozen lung section from a hemizygous *Plvap-T2A-EGFP-IRES-CreERT2* knock-in mouse. Scale bar: 50 μm. **(c)** Representative image of heart section from a hemizygous *Plvap-T2A-EGFP-IRES-CreERT2* knock-in mouse. Scale bar: 50 μm. **(d)** Schematic diagram showing the breeding strategy for generating *Plvap-T2A-EGFP-IRES-CreERT2; Ai14* mice. **(e)** Representative confocal images of kidney and liver sections from *Plvap-T2A-EGFP-IRES-CreERT2; Ai14* mice, showing Ai14-tdTomato expression. Lectin (gray): endothelial profiles. Dapi (blue): nuclear staining. Scale bar: 100 μm. **(f)** A representative image of brain section from a hemizygous *Plvap-T2A-EGFP-IRES-CreERT2* knock-in mouse. Scale bar: 500 μm. **(g)** Representative confocal image showing a whole mount of choroid plexus from a hemizygous *Plvap-T2A-EGFP-IRES-CreERT2*; Ai14-tdTomato mice, 2 weeks after tamoxifen induction. *Plvap-*CreERT2 driven tdTomato *(Plvap-*Cre::Ai14-tdT) are shown in red. Scale bar: 100 μm. **(h)** Representative confocal images showing *Plvap-*Cre::Ai14-tdT and *Plvap-*EGFP expression in the choroid plexus of 3^rd^ ventricle from a brain section. Dapi (blue), nuclei staining. Scale bar: 50 μm. **(i)** Schematic diagram showing the mild traumatic brain injury (mTBI) model with a controlled cortical impact system. **(j)** A whole mount of skull preparation from a mouse 3 days after mTBI injury. Red circle indicates the impacted region. **(k)** Representative confocal images showing *Plvap-*Cre::Ai14-tdT, *Plvap-*EGFP and lectin angiogram (Magenta) in the boxed region of the contralateral side (green box) and ipsilateral side (brown box). Scale bar: 100 μm.

MECA-32 (an antibody against PLVAP glycoprotein) is widely used for staining of endothelial cells in embryos and most adult mouse peripheral tissues^28^, and it also label the vasculature in some of the circumventricular organs, such as the choroid plexus. This is highly consistent with single cell transcriptomic (Fig. 1c). Therefore, we also generated the *Plvap-T2A-EGFP-IRES-CreERT2* knock-in mouse model, carrying the EGFP reporter and CreERT2 recombinase (Fig. 4a). One F0 founder was selected based on germline transmission and genome sequencing, and the F1 generation was further tested with southern blot analysis and PCR for the integrity of the knock-in allele (**Extended Data Fig. 4a-c**). *Plvap-T2A-EGFP-IRES-CreERT2* hemizygous mice are healthy, and we observed robust *Plvap-*EGFP signal in the lung and heart vasculature (Fig. 4b–c), which is consistent with the transcriptomic profiles in the Tabula Muris database^23^ (**Extended Data Fig. 4d**).

To access the CreER activity of *Plvap-T2A-EGFP-IRES-CreERT2* model, we crossed it with the Ai14-tdTomato floxed reporter mice^27^ (Fig. 4d). Following Tamoxifen injections, we analyzed *Plvap-CreERT2* driven Ai14-tdTomato reporter (*Plvap-*Cre::Ai14-tdT), and found very robust labeling of peripheral vasculatures including kidney and liver (Fig. 4e). Interestingly, we also observed *Plvap-*EGFP signals in the choroid plexus within the brain ventricles, but not in the cortex or hippocampus (Fig. 4f). This is further confirmed with the *Plvap-*Cre::Ai14-tdT model, which showed robust labeling of choroid plexus vasculature following Tamoxifen injections (Fig. 4g–h).

In addition, the*Plvap* model also reliably targets the meningeal vessels. As meningeal vasculature is known to be vulnerable in head injuries^29^, we conducted mild traumatic brain injury (mTBI) on this model using a controlled cortical impact system^30^ (Fig. 4i). Even though the impact did not result in direct damage to the skull (Fig. 4j)^30^, the meningeal vessel were severely damaged in the ipsilateral side after the injury, as shown by the dramatic loss of *Plvap-*Cre::Ai14-tdT reporter under the impact site(Fig. 4k). Interestingly, the *Plvap*-EGFP reporter showed an increased pattern 3 days after mTBI, indicating robust angiogenesis and vascular remodeling occurred shortly after the injury(Fig. 4k). This demonstrated the new *Plvap* knock-in model is suitable for studying peripheral endothelial cells, and the built-in reporter and CreERT2 recombinase can be used synchronously for effective lineage tracing *in vivo*.

### Transcriptomic heterogeneity between CNS and peripheral endothelial cells

**Figure 5:**
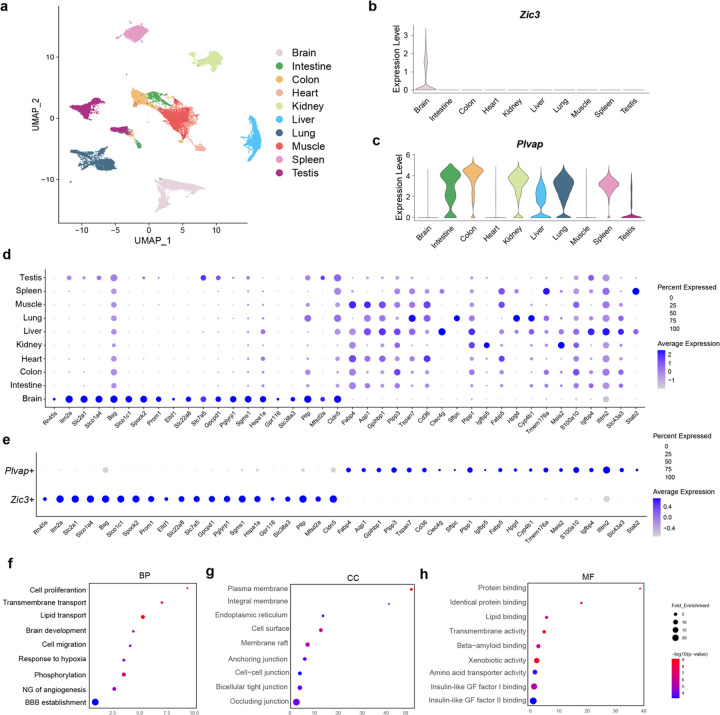
Transcriptomic differences between CNS and peripheral endothelial cells. **(a)** UMAP of single endothelial cells from mouse. Each dot was color-coded and annotated by different organs. **(b)** Violin plot showing the distribution of expression level of *Zic3* in mouse endothelial transcriptomics. **(c)** Violin plots showing the distribution of expression level of *Plvap* in mouse endothelial transcriptomics. **(d)** Dot plots showing the expression of differentially expressed genes across all organs. **(e)** Dot plots showing the expression level of the differentially expressed genes in *Zic3*+ and *Plvap*+ cells, respectively. **(f-h)** GO Pathway analysis on 341 BBB endothelial cells enriched genes. f, Cell proliferation: Positive regulation of cell proliferation; Cell migration: Positive regulation of cell migration; Phosphorylation: Positive regulation of peptidyl−tyrosine phosphorylation; NG of angiogenesis: Negative regulation of angiogenesis; BBB establishment: Establishment of blood-brain barrier. g, Integral membrane: Integral component of membrane. h, Transmembrane activity: Transmembrane transporter activity; Xenobiotic activity: Xenobiotic transporter activity; Amino acid transporter activity: Amino acid transmembrane transporter activity; Insulin like GF factor I binding: Insulin like growth factor I binding; ; Insulin like GF factor II binding: Insulin like growth factor II binding.

To further explore the differences between *Zic3*-positive and *Plvap*-positive endothelial cells at single cell transcriptomic level, we analyzed 37,083 single endothelial cells from various organs, including the brain, intestine, colon, heart, kidney, liver, lung, muscle, spleen, and testis (Fig. 5a–c). Specifically, we compared 1,903 *Zic3*-positive ECs with 20,517 *Plvap*-positive ECs. These two populations exhibit distinct transcriptomic profiles, and we identified 341 genes with significant differential expression: 277 genes are significantly enriched in *Zic3*-positive ECs, while 64 genes show higher expression in *Plvap*-positive ECs. For instance, transporter genes such as *Slc2a1, Slco1a4, Slco1c1, Slc22a8, Slc7a5, Slc38a3 and Mfsd2a* are more abundantly expressed in *Zic3*-positive ECs, whereas lipid metabolism associated genes such as Phospholipid Phosphatases *Plpp1* and *Plpp3*, fatty acid binding protein *Fabp4*, glycosylphosphatidylinositol anchored high density lipoprotein binding protein 1 (*Gpihbp1*) are commonly expressed in *Plvap*-positive ECs (Fig. 5d–e). In addition, a few genes showed organ specificities in the peripheral, e.g. surfactant protein C *(Sftpc)* is only found in lung ECs, C-Type Lectin domain protein *Clec4g* is mainly expressed in liver ECs, Insulin-like growth factor binding protein *Igfbp5* is exclusive to kidney ECs, Stabilin 2 (*Stab2*) is highly enriched in spleen ECs (Fig. 5d). With these differentially expressed genes (DEGs), we conducted pathway analysis based on Gene Ontology enrichment method. Based on biological processes, these DEGs are related to transporter systems and regulate brain development (Fig. 5f). Their cellular functions are connected to membrane functions and intercellular junctions (Fig. 5g), and their molecular functions are associated with lipid binding and protein transporting (Fig. 5h). This was further confirmed with Ingenuity canonical pathway analysis, which showed that transporter system and tight junction signaling are the top pathways differentiating the *Zic3*-positive ECs from *Plvap*-positive ECs (**Extended Data Fig. 5e**). Taken together, these data further confirmed *Zic3* and *Plvap* as unique markers for the CNS and peripheral endothelial cell, respectively.

## Discussion

The mammalian BBB is central to the CNS health and functions. It is an evolutionary milestone for overall brain fitness, yet in same time became a major research challenge, as our understanding of how the highly specialized BBB structure is established during development and how different brain vascular cell types orchestrate together to support brain functions remain limited^10^. This obstacle can be partly attributed to the lack of specific markers and genetic tools that can separate the cerebrovascular cells from the peripheral ones. For example, current murine genetic tools of vascular cells are often based on the well-known endothelial cell markers such as podocalyxin (*Podxl*), claudin 5 (*Cldn5*), tyrosine kinase (*Tek/Tie2*) and tyrosine kinase receptor (*Kdr*), which all tend to express throughout the body. Therefore, transgenic mouse models based on these alleles, including *Tek-Cre* and *Tek-Cre/ERT2, Cdh5-Cre and Cdh5-Cre/ERT2, Kdr-Cre*, all exhibit limitations and constraints when applied to brain vasculature^31^. Recently, we used single cell transcriptomics and identified a brain pericyte specific marker — *Atp13a5*, and successfully generated a knock-in mouse model for the brain pericyte^21^. With similar approaches, we further identified *Zic3* as a brain endothelial specific marker from single cell transcriptomics, and confirmed its expression with both in situ hybridization and antibody staining.

The finding of *Zic3* markers is very meaningful, as it guided us to generate a new transgenic model — *Zic3-T2A-tdTomato-IRES-CreERT2*. Based on the *Zic3-*tdTomato expression and the corresponding CreER recombinase activity after tamoxifen induction, we further confirmed that the *Zic3-T2A-tdTomato-IRES-CreERT2* model is specific to the endothelial cells in the CNS, and not for peripheral cells. For peripheral endothelial cells, we also generated a new *Plvap-T2A-EGFP-IRES-CreERT2* knock-in model, and the characterization of *Plvap*-EGFP reporter and corresponding CreER recombinase activity showed it targets the endothelial cells of the peripheral organs such as lung, kidney, liver, etc., as well as the circumventricular organs in the brain which are known to be outside of the BBB. Therefore, the *Zic3* and *Plvap* genetic tools are sufficient to separate and selectively target the CNS and peripheral endothelial cells, respectively. Interestingly, the presence of *Plvap* in choroid plexus and meningeal vessels also indicates that the heterogeneity of cerebrovascular endothelial cells, at least between the BBB and non-BBB areas, which could be potentially probed with the genetic tools we have generated.

There is a list of receptors and transporters known to be highly enriched at the BBB endothelium, including lysolipid transporter *Mfsd2a*, glucose transporter *Slc2a1* and organic anion transporter *Slco1c1*. Their candidacy for generating genetic models for brain or BBB endothelial cells were proposed, but none of them are specific enough, as shown by single cell transcriptomics (e.g. Extended Data Fig. 1). Based on our data from RNAscope, immunostaining and reporter models, *Zic3* is indeed a CNS endothelial specific genetic marker. *Zic3* encodes a highly conserved C2H2 zinc finger domain protein and a member of the GLI superfamily of transcription factors^32^. Its mutations are found in patients with X-linked heterotaxy, and its deficiency in mice disrupts the left–right asymmetry of cardiovascular development and results in neural tube deficits^33^. While ZIC3 plays an important role in early development, it is still surprising to see its expression remained in the BBB endothelial cells even in the adult brain. In human endothelial cells derived from pluripotent stem cells, ZIC3 can act downstream of Wnt signaling and promote the endothelial barrier functions^34^. Ectopic expression of Zic3 in human umbilical vein endothelial cells could also induce BBB marker gene expression^35^. Therefore, it is important to determine if Zic3 is a master regulator of an intrinsic program that maintains the BBB functions in future studies. Interestingly, *Zic3* is also located on X-chromosome, and may contribute to the sex-difference in the cerebrovascular functions associated with aging and Alzheimer’s disease^36^.

The cerebrovascular development is guided by some of the classic growth factor pathways, such as VEGF, Wnt and Notch^8^. Take Wnt signaling as an example, Wnt7a/7b and Norrin^37^, Frizzled receptor^38^, co-receptors low-density lipoprotein receptor-related protein (LRP) 5 and 6^39^ and co-activator orphan G-protein coupled receptor Gpr124^40^ are all indispensable the proper development of the BBB. To understand the vascular heterogeneity, we analyzed current single cell transcriptomics gathered from different organs. Besides *Zic3*, *Plvap* and *Atp13a5*, we identified a list of genetic markers that can potentially be utilized to develop next-generation tools. For example, the transcriptomic comparison between CNS *Zic3+* endothelial cells with *Plvap* + peripheral ones pointed to hundreds of DEGs that changed preferentially in BBB endothelial cells (Fig. 5). These genes are linked to interesting pathways associated with the establishment of the blood-brain barrier, brain development, lipid metabolism, transport systems and tight junction signaling. While *Zic3* has a preference for CNS endothelial cells, *Clec4g, Igfbp5* and *Stab2* may potentially be utilized to target endothelial cells of the liver, kidney and spleen, respectively, and worth further investigation. Nevertheless, this endothelial heterogeneity is proof that ECs are heavily influenced by the local environment of each individual organs.

Last but not the least, BBB dysfunctions with endothelial activation is commonly found in a spectrum of CNS disorders, including AD and other dementia^10^. Therefore, the identification of *Zic3* as a BBB endothelial cells marker will likely advance our studies towards a better understanding of BBB vascular cell changes in aging and CNS diseases, including vascular contributions to AD and dementia. Beyond the application of these transgenic models, these specific markers can be used to develop other tools, such as BBB cell-type specific antibodies and new viral vector designs to target BBB endothelial cells more specifically with artificial promoters based on tissue and cell-type specific enhancers^41^.

## Methods

### CONTACT FOR REAGENT AND RESOURCE SHARING

Further information and request for resources and reagents should be directed to and will be fulfilled by Lead Contact Zhen Zhao (zzhao@usc.edu).

### EXPERIMENTAL MODEL AND SUBJECT DETAILS

#### Animals

Mice were housed in plastic cages on a 12 h light/dark cycle with access to water ad libitum and a standard laboratory diet. All procedures were approved by the Institutional Animal Care and Use Committee at the University of Southern California and followed National Institutes of Health guidelines. All animals were included in the study. Male and female animals of 2–3 months of age were used in the experiments. All animals were randomized for their genotype information. All experiments were carried out blind: the operators responsible for the experimental procedures and data analysis were blinded and unaware of group allocation throughout the experiments. For all experiments, at least three independent mice were analyzed, which included both sexes and no apparent sex difference were observed.

##### Generation of the Zic3-tdTomato-CreERT2 knock-in model

To generate *Zic3-T2A-tdTomato-IRES-CreERT2 knock-in mouse*, donor DNA templates encoding self-cleaving T2A peptide, tdTomato, internal ribosome entry site and CreERT2 were synthesized. These sequences were flanked by 375bp sequences and 4606bp sequences homologous to the third exon and 3’ UTR region of *Zic3* gene. Next, these donor vector containing the *T2A-tdTomato-IRES-CreERT2* cassette, and gRNA (TTTAACGAATGGTACGTCTGAGG) were co-injected into fertilized eggs to generate targeted conditional knock-in offspring. The F0 founder animals were genotyped by PCR and sequence analysis, and four F1 mice were generated and further confirmed with southern blotting for both 5’ arm and 3’ arm insertion sequences.

##### Generation of the Plvap-EGFP-CreERT2 knock-in model

We generated the *Plvap-T2A-EGFP-IRES-CreERT2* knock-in mouse (Fig. 4b), with a donor DNA template encoding self-cleaving T2A peptide, EGFP, internal ribosome entry site, CreERT2. These sequences were flanked by 405bp sequences and 3834bp sequences homologous to the 6^th^ exon and 3’ UTR region of *Plvap* gene. These donor vector containing the *T2A-EGFP-IRES-CreERT2* cassette, and gRNA (GCAGCTGGGTCCTCAACCGCTGG) were co-injected into fertilized eggs to generate targeted conditional knock-in offspring. The F0 founder animals were genotyped by PCR and sequence analysis, and three F1 mice were generated and further confirmed with southern blotting for both 5’ arm and 3’ arm insertion sequences.

##### Mild Traumatic Brain Injury model

To induce mild traumatic brain injury (mTBI) in mice, we followed a previously described protocol^42^. Briefly, we used the KOPF stereotaxic system to position the mouse’s head under the impactor at a specific angle, targeting a point 2 mm posterior and 2.5 mm lateral to Bregma. A 4 mm flat plastic tip (RWD Life Science) was used to deliver a controlled impact using a brain injury device (RWD #68099). Mice were anesthetized with ketamine and xylazine (90 mg/kg and 9 mg/kg, i.p.). After exposing the skull, we delivered an impact at a velocity of 3 m/s, a depth of 1 mm, and a duration of 180 milliseconds. Mice were then placed in warmed cages to recover.

### METHOD DETAILS

#### Bioinformatics

##### ScRNA-seq data for mouse brain vasculature and multiple organs

For scRNA-seq integration dataset, we obtained the cell count matrix from Gene Expression Omnibus (GEO) with the series record GSE98816, GSE150294 and Array express E-MTAB-8077, and did secondary analysis after integration. Then we performed secondary analysis of 3 single cell sequencing (scRNA-seq) datasets on brain and peripheral vascular cells, 48,526 single-cell transcriptomes were collected using R Seurat Package.

For scRNA-seq dataset for mouse brain vasculature, we obtained the cell count matrix from GEO with the series record GSE98816 and GSE99058^18^. The data represent the expression levels of 18435 genes in 3186 cells. The mouse brain tissue was harvested for Smart-seq2 and sequencing was performed on a HiSeq2500 at the National Genomics Infrastructure (NGI), Science for Life Laboratory, Sweden, with single 50-bp reads (dual indexing reads).

For scRNA-seq dataset for multiple organs, we obtained the cell count matrix from GEO with the series record GSE109774^23^. The data represent the expression levels of 23433 genes in 53760 cells. All organs were single-cell-sorted into plated using flurescence-activated cell sorting. Libraries were sequenced on the NovaSeq 6000 Sequencing System (Illumina) using 2 × 100-bp paired-end reads.

##### ScRNA-seq data preprocessing

The data processing of the scRNA-seq data were performed with the Seurat Package (v.3.1.5) in R (v.3.6.2). The basic scRNA-seq analysis was run using the pipeline provided by Seurat Tutorial (https://satijalab.org/seurat/v3.0/immune_alignment.html) as of June 24, 2019. In general, we set up the Seurat objects from different groups in experiments for normalizing the count data present in the assay.

This achieves log-normalization of all datasets with a size factor of 10,000 transcript per cell. For different Seurat objects, FindVariableFeatures() function was used to identify outlier genes on a ‘mean variability plot’ for each object. The nFeatures parameter is 2000 as the default for the selection method called ‘vst’. These resulted genes serve to illustrate priority for further analysis.

##### Data processing

The dataset on all cells were used to scale and center the genes. First of all, principal component analysis (PCA) was used for linear dimensionality reduction with default computes the top 30 principal components. By applying the JackStraw() function, JackStrawPlot() function and ElbowPlot() function, we identified the principal components for further analysis. Then, PCA results were used as the input for the Uniform Manifold Approximation and Projection (UMAP) dimensional reduction.

We identified clusters of cells by a shared nearest neighbor (SNN) modularity optimization-based clustering algorithm. The algorithm first calculated k-nearest neighbors and computed the k-NN graph, and then optimizes the modularity function to determine clusters.

##### Determination of cell-type identity

To determine the cell type, we used FindAllMarkers() function with parameters min.pct and thresh.use set to 0.25 to find markers in each cluster and known marker genes that have been previously reported could be used to determine cell-type identity. These include, but are not limited to *Snap25* for Neuron, *Cldn10* for Astrocyte, *Mbp* for Oligodendrocyte, *Cldn5* for EC, *Kcnj8* for PC, *Acta2* for VSMC, *Ctss* for microglial, *Col1a1* for Fibroblast-like cell.

##### Pathway analysis and visualization by Metascape, ClueGO and Cytoscape

Using the Metascape online tool (http://metascape.org), we performed functional enrichment analysis of ZIC3-positive enriched genes. Enrichment of pathways from KEGG, GO Biological Process and GO Molecular Function was analyzed by Metascape. The terms with P-value < 0.01, minimum counts of 3, and enrichment factors of > 1.5 would be considered. The ClueGO Cytoscape pluin 2.5.4 and Cytoscape version 3.8.1 will be used for secondary KEGG pathway analysis and network visualization.

#### Cellular Biology Related Procedures

##### HPR injection and Lectin injection and mapping

The HRP solution was prepared by dissolving 125 mg (0.125 g) of HRP Type II (Sigma, P8250) in 2.5 ml of PBS, yielding a concentration of 0.5 mg/10 μl. Each animal was injected with a single dose of 0.5 mg/g of body weight and harvested 2 hours later. The mice were then sacrificed and perfused with PBS and PFA at 5 min after lectin (ThermoFisher Scientific, #L32470) injection. To visualize HRP in the injected samples under light microscopy, the samples were washed with PBS and incubated in Tris buffer containing 0.1% tyramide reagent and 0.0015% H_2_O_2_ for 10 minutes at room temperature, in the dark^43^. All sections were then scanned using the Li-Cor Odyssey Dlx at a resolution of 21 μm, or on a NikonTi2 confocal microscope. The ImageJ plugin ‘Neuro J’ length analysis tool was used to measure the length of lectin-positive or HRP-positive endothelial capillary profiles. The capillary length was quantified and expressed as mm of lectin+ endothelial capillary profiles per mm2 of brain tissue. The HRP-occupied vascular area ratio was calculated by measuring the HRP-positive signal within lectin-positive regions.

#### Fluorescence *in situ* hybridization

Fluorescence *in situ* hybridization was performed using the RNAscope technology (Advanced Cell Diagnostics, Hayward, CA). Tissue sample preparation and pretreatment were performed on fixed brains cut into 15 μm sections mounted onto SuperFrost Plus glass slides following the manufacturer’s protocol (ACD documents 323100). After dehydration and pretreatment, slides were subjected to RNAscope Multiplex Fluorescent Assay (ACD documents 323100). RNAscope probes for mouse *Zic3,* positive control and negative control were hybridized for 2h at 40°C in the HybEZ Oven and the remainder of the assay protocol was implements. Subsequently, the slides were subjected to immunohistochemistry. The fluorescent signal emanating from RNA probes and antibodies was visualized and captured using a Nikon AIR MP+ confocal/ multiphoton microscope (Nikon). All FISH images presented are projection of 10-image stacks (0.5 μm intervals) obtained from cerebral cortex, and a smoothing algorithm was applied during image post-processing (Nikon NIS-Elements Software).

#### Immunohistochemistry

Animals were anesthetized, perfused and brains were removed and postfixed as we described previously^44^. Brain, spinal cord, kidney, liver, and heart tissue were also collected, postfixed and cut at 35 μm thickness using a vibratome (Leica). After that, sections were blocked with 5% normal donkey serum (Vector Laboratories) and 0.1% Triton-X in 0.01M PBS and incubated with primary antibodies diluted in blocking solution overnight at 4°C. The primary antibody information is as following: Goat anti-mouse aminopeptidase N/ANPEP (CD13; R&D systems; AF2335; 1:100), ZIC3 polyclonal antibody (Invitrogen; PA5–29073; 1:100), Rat anti-mouse vascular adhesion molecule (VCAM1; MilliporeSigma; CBL1300; 1:200), Mouse anti-α-smooth muscle actin (SMA, MilliporeSigma; A5228, 1:200), Rabbit anti-mouse ionized calcium binding adaptor molecule 1 (Iba-1; Wako, 019–19741; 1:200), Rabbit anti-Glial Fibrillary Acidic Protein (GFAP; Dako, z0334; 1:500), Rabbit anti-mouse NeuN (Millipore, ABN78, 1:500). To visualize brain microvessels, sections were incubated with Dylight 488 or 647-conjugated *L. esculentum* lectin as we have described previously^44^. After incubation with primary antibodies, sections were washed with PBS for three times and incubated with fluorophore-conjugated secondary antibodies. Sections were imaged with a Nikon AIR MP+ confocal/ multiphoton microscope (Nikon). Z-stack projections and pseudo-coloring were performed using Nikon NIS-Elements Software. Image post analysis was performed using ImageJ software.

#### Molecular Biology Related Procedures

##### DNA isolation and genotyping

Mouse genomic DNA was isolated from tail biopsies (2 – 5 mm) and following overnight digestion at 56 into 100 μL of tail digestion buffer containing 10 mM Tris-HCl (pH 9.0), 50 mM KCl, 0.1% Triton X-100 and 0.4 mg/mL Proteinase K. Next, the tail will be incubated at 98 for 13 minutes to denature the Proteinase K. After centrifugation at 12000 rpm for 15 min, the supernatants were collected for PCR. The primers details are listed in the KEY RESOURCES TABLE. The PCR conditions were as follows: 1) 94 °C for 3 min; 2) 35 cycles at 94 °C for 30 sec, 60 °C for 30 sec, and 72 °C for 35 sec; 3) 72 °C for 5 min. PCR products were separated on 2% agarose gel.

##### Quantification and statistical analysis

Sample sizes were calculated using nQUERY, assuming a two-side alpha-level of 0.05, 80% power and homogeneous variances for the 2 samples to be compared, with the means and SEM for different parameters predicted from pilot study. All the data are presented as mean ± SEM as indicated in the figure legends and were analyzed by GraphPad Prism 8. For multiple comparisons, Bartlett’s test for equal variances was used to determine the variances between the multiple groups and one-way analysis of variance (ANOVA) followed by Tukey test was used to test statistical significance, using GraphPad Prism 8 software. A P value of less than 0.05 was considered statistically significant.

## Supplementary Files

This is a list of supplementary files associated with this preprint. Click to download.

- ExtendedFigures.docx

## Figures and Tables

**Table:** KEY RESOURCES TABLE

REAGENT or RESOURCE	SOURCE	IDENTIFIER
**Genomic data sets**
Mouse brain vasculature	Gene Expression Omnibus	GSE98816 GSE99058
Atlas of Murine Endothelial Cells	Array Express	E-MTAB-8077
Fibroblast and mural cell in muscular organs	Gene Expression Omnibus	GSE150294
Tabula Muris	Gene Expression Omnibus	GSE109774
**Single cell RNA-sequencing data processing**
Seurat Package (v.3.1.5)	R	v.3.6.2
FindVariableFeatures function	R	v.3.6.2
FindAllMarkers function	R	v.3.6.2
FindMarkers function	R	v.3.6.2
Principal component analysis (PCA)	R	v.3.6.2
Uniform Manifold Approximation and Projection (UMAP)	R	v.3.6.2
Shared Nearest Neighbor (SNN) clustering	R	v.3.6.2
**Experimental Models: Organisms/Strains**
*Zic3-tdTomato-CreERT2*	This study	
*Plvap-tdTomato-CreERT2*	This study	
***DNA isolation and genotyping***
GoTaq Green Master Mix	Promega	M7122
*Zic3-tdTomato-*CreERT2 forward primer	AATGGCTCTCCTCAAGCGTATTC	
*Zic3-tdTomato-*CreERT2 reverse primer	GTTATTCAACTTGCACCATGCCG	
*Plvap-EGFP-*CreERT2 forward primer	GCTGTGTAGCAGAGACAAACCTTA	
*Plvap-EGFP-CreERT2* reverse primer	GGTGGTGCAGATGAACTTCAGG	
**Antibodies**
Goat polyclonal anti-CD13	R&D systems	AF2335
Rat monoclonal anti- CD31	BD Pharmingen	550274
Rabbit polyclonal anti-ZIC3	Thermo Fisher Scientific	PA5–29073
Rat monoclonal anti-VCAM1	Millipore Sigma	CBL1300
Mouse monoclonal anti-SMA	Millipore Sigma	A5228
Rabbit polyclonal anti-Iba-1	Wako	019–19741
Rabbit polyclonal anti-GFAP	Dako	z0334
Rabbit polyclonal anti-NeuN	Millipore Sigma	ABN78
Rabbit anti-mouse Olig2	Millipore Sigma	AB9610
Dylight 488-conjugated *L. esculentum* lectin	Thermo Fisher Scientific	L32470
Dylight 649-conjugated *L. esculentum* lectin	Thermo Fisher Scientific	L32472
**Immunohistochemistry**
Vibratome	Leica	VT1200
Cryostat	Leica	CM3050 S
Normal Donkey Serum	Jackson ImmunoResearch	AB_2337258
VECTASTAIN ABC universal kit	Vector laboratories	PK6200
ImmPRESS Universal Polymer kit	Vector laboratories	MP-7500
***in situ* hybridization**
SuperFrost Plus Micro Slide	VWR	48311–703
*Zic3* RNAscope probe	Advanced Cell Diagnostics	Cat. #480351
Positive control RNAscope probe	Advanced Cell Diagnostics	Cat. #320881
Negative control RNAscope probe	Advanced Cell Diagnostics	Cat. #320871
HybEZ Oven	Advanced Cell Diagnostics	
RNAscope^®^ 2.5 HD Reagent Kit-RED	Advanced Cell Diagnostics	322350
RNAscope^®^ Multiplex Fluorescent V2 Assay	Advanced Cell Diagnostics	323100
Nikon A1R MP+ confocal/ multiphoton microscope	Nikon	Nikon A1R
Revolve 4 brightfield and fluorescence microscope	Echo	RVL-100-G
**Software**
GraphPad Prism	GraphPad Software	GraphPad Prism 8
Metascape	http://metascape.org	
ClueGO		2.5.4
Cytoscape		3.8.1
Image J	NIH	
